# Effectiveness of acupuncture for the treatment of postoperative pain: A protocol for a systematic review of randomized controlled trial

**DOI:** 10.1097/MD.0000000000017606

**Published:** 2019-12-10

**Authors:** Qinhong Zhang, Jin-Huan Yue, Zhong-Ren Sun, Brenda Golianu

**Affiliations:** a Department of acupuncture and moxibustion, Heilongjiang University of Chinese Medicine, Harbin, China; b Neuro Acupuncture Health Center, Fremont; c University of Herbal Medicine, Hayward; d Department of Anesthesia, Stanford University, CA.

**Keywords:** acupuncture, effectiveness, postoperative pain, randomized controlled trial, safety

## Abstract

**Background:**

This aim of this study is to assess the effectiveness and safety of acupuncture for the treatment of patients with postoperative pain (PPP).

**Methods:**

We will carry out a systematic review of the published literature and will comprehensively search Cochrane Library, MEDLINE, EMBASE, CINAHL, PsycINFO, Allied and Complementary Medicine Database, Chinese Biomedical Literature Database, and China National Knowledge Infrastructure from inception to the present with no language restrictions. Randomized controlled trials comparing acupuncture with other interventions or sham acupuncture will be included. Two reviewers will independently conduct study selection, data collection, and study quality. A third reviewer will resolve any discrepancies. We will apply RevMan 5.3 software for statistical analysis.

**Results:**

The protocol of this study will systematically assess the effectiveness and safety of acupuncture for patients with PPP. The primary outcome is postoperative pain intensity. The secondary outcomes comprise of: analgesic consumption, postoperative recovery parameters, vital signs, quality of life, and treatment related adverse events.

**Conclusion:**

This study will summarize the current evidence base for the effectiveness and safety of acupuncture for patients with PPP.

## 1 Introduction

Postoperative pain (PPP) is one of the most frequent symptoms encountered by patients following surgery.^[1–3]^ It has been estimated that about 86% of surgery patients experience moderate to severe PPP.^[4]^ In addition, more than 50% of these patients still experience persistent chronic PPP.^[5]^ These conditions may also restrict physical activity, prolong recovery time, and affect quality of life.^[6–7]^ Furthermore, PPP may also lead to postoperative complications, increase postoperative morbidity, extend hospital stay, and increase health care costs.^[8–9]^

Acupuncture has been demonstrated to be useful in the management of a variety of pain disorders, including headache, migraine, neck pain, shoulder pain, elbow pain, back pain, hip pain, knee pain, leg pain, and ankle pain.^[10–18]^ In addition, numerous randomized clinical trials have also reported that it can effectively decrease PPP.^[19–31]^ However, to date no study has systematically explored its effectiveness and safety for patients with PPP. Thus, this study will aim to systematically assess the effectiveness and safety of acupuncture for patients with PPP.

## 2 Methods and analysis

### 2.1 Ethics and dissemination

Ethical approval is not needed, because individual data will not be involved. This study will be published in a peer-reviewed journal.

### 2.2 Study inclusion and exclusion criteria

#### 2.2.1 Types of studies

All randomized controlled trials (RCTs) of the application of acupuncture in the treatment of patients with PPP will be included with no language limitation. However, animal studies, case reports, case series, commentaries, reviews, non-controlled trials, and non-RCTs will be excluded.

#### 2.2.2 Types of interventions

The participants in the intervention group have received acupuncture treatment alone, in addition to standard medical care.

The patients in the control group have received other interventions or sham acupuncture in addition to the same standard medical care as the intervention group.

#### 2.2.3 Types of participants

We will include patients diagnosed with PPP with no limitations of race, gender, and age.

#### 2.2.4 Types of outcome measurements

The primary outcome is pain intensity, which can be measured by numerical rating scales or any other scales.

The secondary outcomes include analgesic consumption, post-operative recovery parameters, vital signs, quality of life, and treatment related adverse events.

### 2.3 Search methods for the identification of studies

#### 2.3.1 Electronic database searches

With the assistance of a librarian, we will comprehensively search relevant literature from Cochrane Library, MEDLINE, EMBASE, CINAHL, PsycINFO, Allied and Complementary Medicine Database, Chinese Biomedical Literature Database, and China National Knowledge Infrastructure from inception to the present with no language restrictions. We will include RCTs on assessing effectiveness and safety of acupuncture for the treatment of patients with PPP. The retrieval strategy for Cochrane Library is showed in Table 1. In addition, similar search strategy will be adapted to other electronic databases.

**Table 1:**
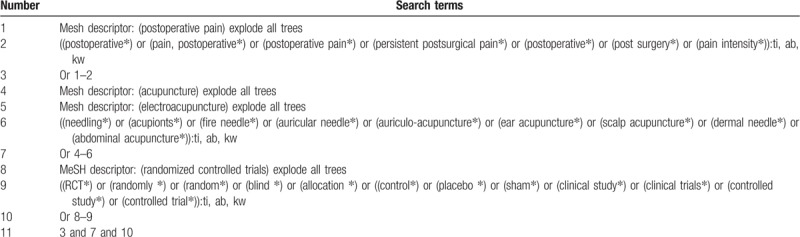
Search strategy sample of Cochrane Library.

#### 2.3.2 Other literature sources search

We will also search dissertations, conference proceedings, and reference lists of relevant included studies.

### 2.4 Data collection and analysis

#### 2.4.1 Study selection

**Figure 1:**
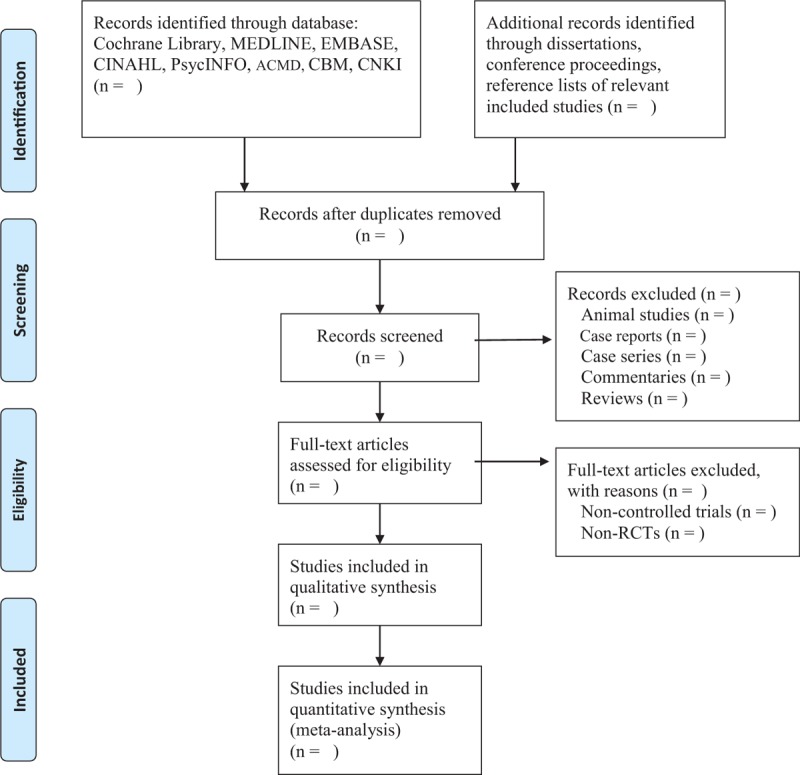
Flowchart of study selection.

After retrieving initial results by scanning titles and abstracts of all records, irrelevant studies and duplicated studies will be removed. After that, the full-texts of the remaining studies will be further evaluated according to the previously described inclusion criteria. Two authors will independently carry out study selection. A third author will be consulted where consensus is not reached between 2 authors. The flowchart of study selection process will be showed in Figure 1.

#### 2.4.2 Data extraction and management

All data will be extracted based on the customized data extraction form by 2 independent authors. Any conflicts between 2 authors will be solved by discussion with the help of a third author where consensus is not reached. Variables to be extracted include the following information: study data (first author, year of publication, study type, country, journal, study setting, etc), patient data (age, gender, diagnosis criteria, eligibility criteria, etc), study methods (randomization, concealment, blinding, etc), intervention details (treatment types, dosage, frequency, etc), and outcome measurements (primary and secondary outcomes, adverse events, follow-up results, etc).

#### 2.4.3 Missing data dealing with

Any unclear or missing data from eligible studies will be inquired from primary authors using email. We will analyze available data if those data cannot be obtained.

### 2.5 Study quality assessment

Methodological quality of all eligible RCTs will be evaluated using Cochrane risk of bias tool for evaluating risk of bias. There are 7 domains, and each domain is further judged as low, unclear, and high risk of bias.

### 2.6 Measures of treatment effect

Mean difference or standard mean difference and 95% confidence intervals will be utilized as the effect measures of continuous variables. For dichotomous values, Risk ratio and 95% confidence intervals as the effect measures of binary variables will be presented.

### 2.7 Assessment of heterogeneity

Heterogeneity will be estimated by *I*^2^ test among included studies. When the value of *I*^2^ is 50% or less, heterogeneity is acceptable, and a fixed-effects model will be utilized for data synthesis. Otherwise, when the value of *I*^2^ is more than 50%, heterogeneity is high, and a random-effects model will be used for data synthesis.

### 2.8 Statistical analysis

RevMan 5.3 software will be applied for statistical analysis. If the heterogeneity is acceptable, meta-analysis will be performed. If the heterogeneity is significant, it is inappropriate to carry meta-analysis, and subgroup analysis will be conducted. If there is still such substantial heterogeneity among those included studies, we will not pool the data and provide a narrative summary instead of meta-analysis.

### 2.9 Additional analysis

#### 2.9.1 Subgroup analysis

We will investigate whether heterogeneity resulted from other confounders, such as different characteristics, treatments and compactors, and outcomes.

#### 2.9.2 Sensitivity analysis

We will assess the robustness of outcome results by excluding studies with high risk of bias.

#### 2.9.3 Reporting bias

In order to detect reporting bias, funnel plot and Egger regression test will be used for quantitative assessments if more than 10 eligible RCTs are included.^[32–33]^

## 3 Discussion

The current literature maintains that acupuncture is effective at decreasing PPP in postoperative patients. However, the evidence for the effectiveness improvement of postoperative recovery and safety for patients with PPP is still inconclusive. Therefore, this study will aim to systematically and comprehensively search literature records. It will address a new aspect related to published studies to explore the effectiveness and safety of acupuncture for PPP. Its results will provide the latest evidence of acupuncture for PPP in both clinical practice and to further research in the field.

## Author contributions

**Conceptualization:** Qinhong Zhang, Jinhuan Yue, Zhongren Sun.

**Data curation:** Qinhong Zhang, Jinhuan Yue, Zhongren Sun.

**Formal analysis:** Qinhong Zhang, Jinhuan Yue.

**Funding acquisition:** Qinhong Zhang, Zhongren Sun.

**Investigation:** Zhongren Sun.

**Methodology:** Qinhong Zhang, Jinhuan Yue, Zhongren Sun.

**Project administration:** Zhongren Sun.

**Resources:** Qinhong Zhang, Jinhuan Yue.

**Software:** Qinhong Zhang, Jinhuan Yue.

**Supervision:** Zhongren Sun.

**Validation:** Qinhong Zhang, Jinhuan Yue, Zhongren Sun.

**Visualization:** Qinhong Zhang, Jinhuan Yue, Zhongren Sun.

**Writing – original draft:** Qinhong Zhang, Jinhuan Yue, Zhongren Sun.

**Writing – review & editing:** Qinhong Zhang, Jinhuan Yue.
